# Pathogenic residue insertion in neuronal nicotinic receptor alters intra- and inter-subunit interactions that tune channel gating

**DOI:** 10.1016/j.jbc.2024.107266

**Published:** 2024-04-06

**Authors:** Deborah J. Msekela, Steven M. Sine

**Affiliations:** 1 Department of Molecular Pharmacology and Experimental Therapeutics, Mayo Clinic College of Medicine and Science, Rochester, Minnesota, USA; 2 Department of Physiology and Biomedical Engineering, Mayo Clinic College of Medicine and Science, Rochester, Minnesota, USA; 3 Department of Neurology, Mayo Clinic College of Medicine and Science, Rochester, Minnesota, USA

**Keywords:** neuronal nicotinic receptor, epilepsy, single-channel patch clamp electrophysiology, single-channel open time, channel gating

## Abstract

We describe molecular-level functional changes in the α4β2 nicotinic acetylcholine receptor by a leucine residue insertion in the M2 transmembrane domain of the α4 subunit associated with sleep-related hyperkinetic epilepsy. Measurements of agonist-elicited single-channel currents reveal the primary effect is to stabilize the open channel state, while the secondary effect is to promote reopening of the channel. These dual effects prolong the durations of bursts of channel openings equally for the two major stoichiometric forms of the receptor, (α4)_2_(β2)_3_ and (α4)_3_(β2)_2_, indicating the functional impact is independent of mutant copy number per receptor. Altering the location of the residue insertion within M2 shows that functionally pivotal structures are confined to a half turn of the M2 α-helix. Residue substitutions within M2 and surrounding α-helices reveal that both intrasubunit and intersubunit interactions mediate the increase in burst duration. These interactions impacting burst duration depend linearly on the size and hydrophobicity of the substituting residue. Together, the results reveal a novel structural region of the α4β2 nicotinic acetylcholine receptor in which interhelical interactions tune the stability of the open channel state.

An enduring goal in medical physiology is to relate form to function in normal and pathological conditions. In this context, the field of membrane proteins, including synaptic receptors, is poised for major advances. Over the past decade the application of cryo-EM has greatly increased the inventory of high-resolution structures, providing concrete starting points to relate form to function. However vivid these structures may be, understanding how the individual parts contribute to function requires complementary experimental approaches. A powerful complementary approach is to study naturally occurring pathogenic variants, which inherently target functionally pivotal structures. Studies of these pivotal structures not only offer the potential to delineate structural mechanisms underpinning function but they also provide bases to develop therapeutic solutions.

Naturally occurring variants of the nicotinic acetylcholine receptor (nAChR) were discovered nearly 3 decades ago (1, 2), with a missense variant of the muscle nAChR being the first shown to cause aberrant function in both native and cloned receptors using single-channel patch clamp electrophysiology (2). In that case, change of a single nucleotide in the gene encoding the ε-subunit converted a Thr residue to Pro within the M2 α-helix that lines the ion conductance pathway, causing aberrant channel opening in the absence of agonist and prolonged channel opening in its presence. The M2 α-helix thus emerged as pivotal in setting both basal and agonist-activated channel activity. These studies also provided a rationale for developing therapy in which a long-lived channel-blocking drug countered the prolonged channel openings (3). Since then, most pathogenic missense variants of the nAChR have been shown to arise from change of a single nucleotide altering one amino acid residue, whereas in-frame nucleotide insertions or deletions are rare (4, 5, 6). From a structure-function standpoint, residue insertions are novel not only because they alter inter-residue interactions at the site of the insertion but also because they alter interactions involving flanking residues.

Here, we investigate functional consequences of a pathogenic residue insertion in the neuronal nicotinic α4β2 nAChR, α4^259Leu260^, originally discovered in a patient with sleep-related hyperkinetic epilepsy (7). When mapped upon the structure of the α4β2 nAChR (8), the pathogenic Leu insertion localizes to the region of the M2 transmembrane α-helix near the extracellular side of the cell membrane, with the hydrophobic Leu side chain projecting into the bundle of α-helices within the α4 subunit facing away from the pore (Fig. 1). The inserted Leu residue takes the place of the native Ile260 residue, altering inter-residue interactions locally, and advancing adjacent residues toward the pore and the linker spanning the M2 and M3 α-helices.

**Figure 1:**
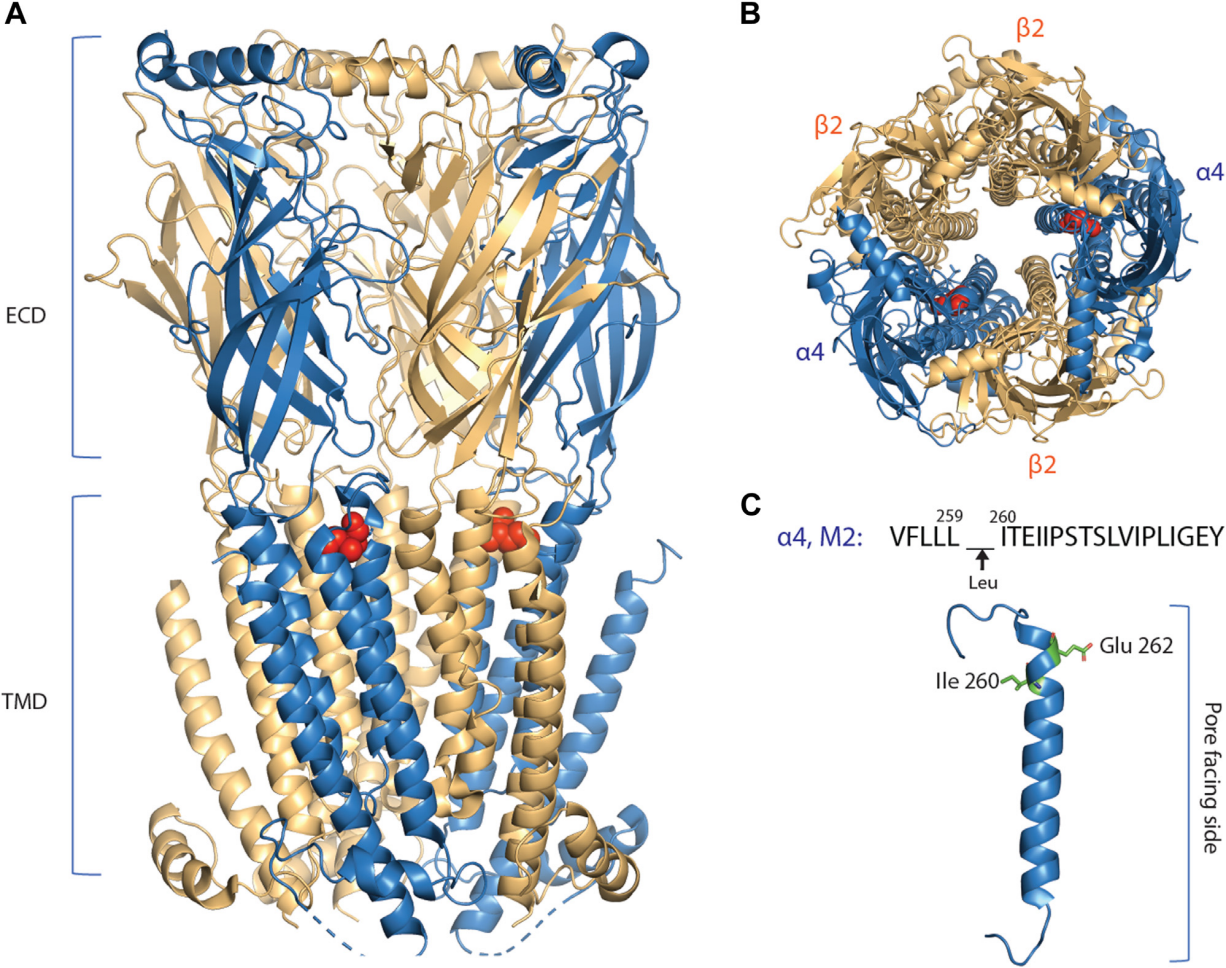
**Structure of the (α4)**_**2**_**(β2)**_**3**_**nAChR (PDB:****6CNJ****) and location of the pathogenic α4**^**259Leu260**^**residue insertion.***A*, *side view* of the receptor is shown in *cartoon representation* with the α4 subunits in *blue*, β2 subunits in *beige*, and the Ile260 that is displaced by the Leu insertion is indicated by *red spheres*. Extracellular and transmembrane domains are labeled ECD and TMD, respectively. *B*, *top view* of the receptor. *C*, *side view* of the M2 domain from the α4 subunit showing Ile260 adjacent to the pathogenic Leu insertion and Glu262 facing the pore in *stick representation*. Note the pathogenic Leu insertion faces away from the pore. nAChR, nicotinic acetylcholine receptor.

The pathogenic α4^259Leu260^ insertion was shown to increase agonist sensitivity when expressed in a heterologous expression system and studied *via* whole-cell macroscopic current recording (7). However, when measured *via* whole-cell macroscopic currents, increases in agonist sensitivity do not distinguish between changes in agonist binding affinity and changes in gating of the ion channel (9) nor do they distinguish between effects on the different stoichiometric forms of the α4β2 nAChR (10, 11). To overcome these limitations, the present study uses single-channel patch clamp electrophysiology to elucidate mechanisms behind the α4^259Leu260^ variant. In addition, to delineate the multifaceted structural effects of the residue insertion, we use a series of engineered residue insertions and substitutions to identify local structural interactions crucial for tuning gating of the ion channel.

## Results

**Figure 2:**
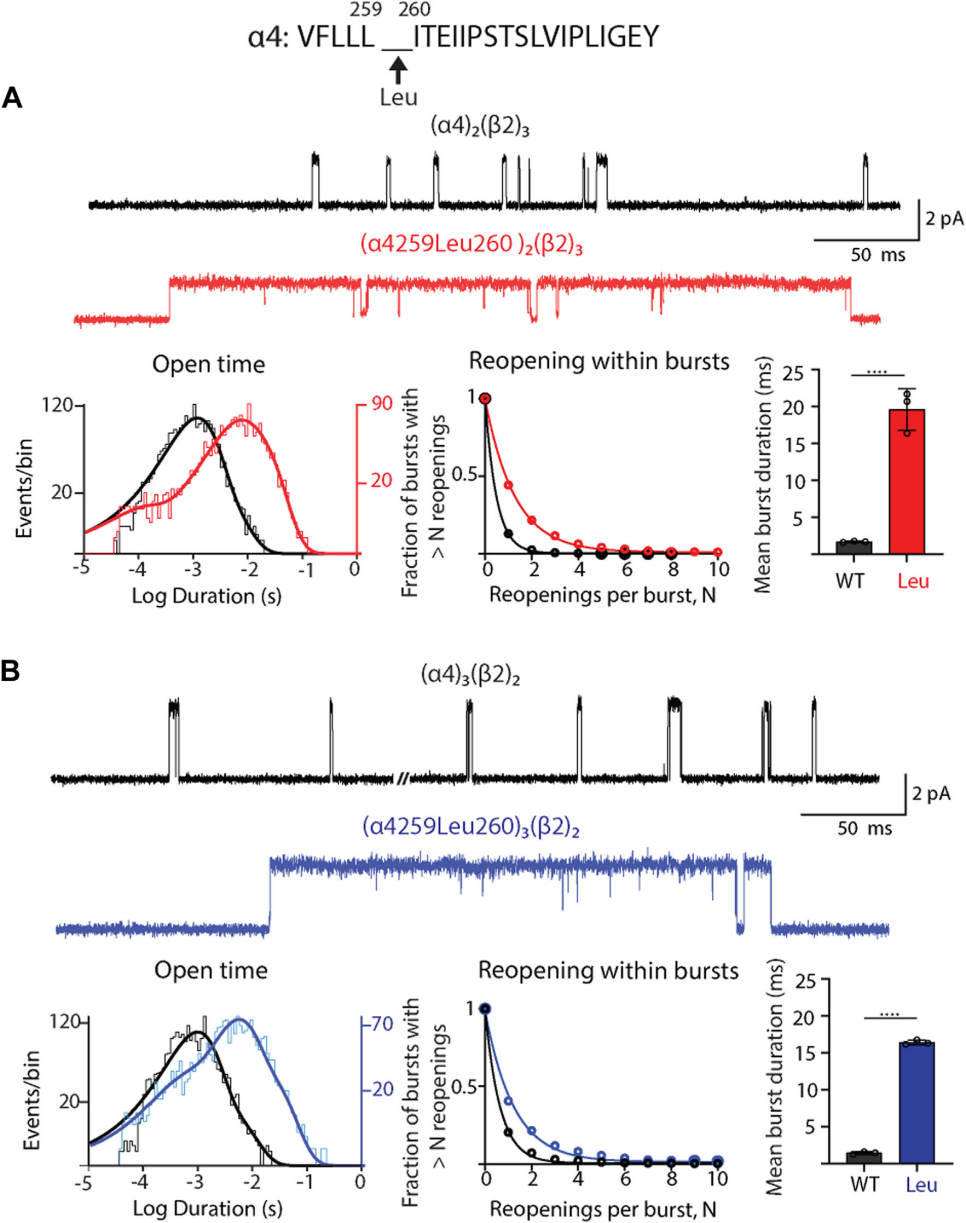
**Single-channel recordings from WT and pathogenic variant nAChRs of the indicated stoichiometric forms activated by ACh.***A*, recordings from the (α4)_2_(β2)_3_ stoichiometry for WT *black trace* and pathogenic variant *red trace* in the presence of 1 μM ACh. Corresponding open time histograms fitted by the sum of exponentials are shown underneath the traces. Probability of channel reopening *versus* the number of reopenings per burst is fitted by a single exponential decay for WT and pathogenic variant receptors. *Bar graph* compares the mean duration of all bursts for WT and pathogenic variant receptors; *circles* indicate individual determinations. *B*, data for the (α4)_3_(β2)_2_ stoichiometry, as in *panel A*, with results for the pathogenic variant shown in *blue*. The recordings in *A* and *B* were obtained using the cell-attached patch configuration at a membrane potential of −70 mV and displayed at 5 kHz bandwidth. N = 3 recordings per mutant for burst duration analysis. An unpaired *t* test was used to obtain ∗∗∗∗*p* < 0.0001 for *panels A* and *B*. See Tables 2 and 3 for reopening decay constants and mean burst durations. nAChR, nicotinic acetylcholine receptor.

To delineate mechanisms behind the gain of function caused by the pathogenic variant, α4^259Leu260^, we employed single-channel electrophysiology together with structural changes *via* site-directed mutagenesis. To begin, we expressed cDNAs encoding α4^259Leu260^ and WT β2 subunits in Bosc 23 cells, a clonal human fibroblast cell line (Experimental procedures). We then used the patch clamp technique to record single ion channel currents elicited by a low concentration of ACh for each stoichiometric form of WT and pathogenic variant receptors; each stoichiometric form was selectively expressed using a different chaperone, as described (12). The recordings reveal markedly prolonged channel opening episodes by the pathogenic variant compared to its WT counterpart for each stoichiometric form (Fig. 2, *A* and *B*). Histograms of open durations show a marked shift toward increased duration for the pathogenic variant compared to WT. To further quantify changes in open duration, we computed the mean duration of all openings for each stoichiometric form, which again reveals marked increases for the pathogenic variant compared to WT (Table 1). Thus, the pathogenic variant stabilizes the open state of the receptor channel of each stoichiometric form.

**Table 1:** Effect of α4 variants on mean channel open time

Position of inserted residue	Residue	Receptor type (number of patches)	Mean open time (ms) (95% CI)	*p* value for WT *versus* mutant	Fold change for mutant relative to WT
	WT	(α4)_2_(β2)_3_ (n = 3)	1.36 (1.32–1.401)	-	-
259–260	Leu	(α4^259Leu260^)_2_(β2)_3_ (n = 3)	9.59 (9.12–10.1)	0.0005	7.05
	Ile	(α4^259Ile260^)_2_(β2)_3_ (n = 3)	5.11 (4.88–5.34)	0.0487	3.76
	Val	(α4^259Val260^)_2_(β2)_3_ (n = 3)	6.99 (6.70–7.29)	0.0063	5.14
	Ala	(α4^259Ala260^)_2_(β2)_3_ (n = 3)	9.437 (7.7–11.26)	0.0005	6.94
	WT	(α4)_3_(β2)_2_ (n = 3)	1.18 (1.12–1.24)	-	-
	Leu	(α4^259Leu260^)_3_(β2)_2_ (n = 3)	7.92 (1.47–8.36)	0.0003	6.70
260–261	Leu	(α4^260Leu261^)_2_(β2)_3_ (n = 3)	1.65 (1.60–1.712)	0.9883	1.21
	Ile	(α4^260Ile261^)_2_(β2)_3_ (n = 3)	4.60 (4.39–4.81)	0.0388	3.28
	Ala	(α4^260Ala261^)_2_(β2)_3_ (n = 3)	11.0 (10.1–11.9)	<0.0001	8.09
261–262	Val	(α4^261Val262^)_2_(β2)_3_ (n = 3)	2.06 (1.96–2.16)	0.1630	1.51
	Ala	(α4^261Ala262^)_2_(β2)_3_ (n = 3)	2.13 (2.07–2.20)	0.1570	1.57
262–263	Leu	(α4^262Leu263^)_2_(β2)_3_ (n = 3)	1.47 (1.38–1.55)	0.9717	1.08
	Ala	(α4^262Ala263^)_2_(β2)_3_ (n = 3)	2.73 (2.64–2.82)	0.0500	2.01
268–269	Ala	(α4^268Ala269^)_2_(β2)_3_ (n = 3)	0.612 (1.56–0.66)	<0.0001	0.45
270–271	Ala	(α4^270Ala271^)_2_(β2)_3_ (n = 3)	0.954 (0.90–1.01)	0.0013	0.701
Substitutions					
261	Leu	(α4^Thr261Leu^)_2_(β2)_3_ (n = 3)	10.9 (10.5–11.5)	<0.0001	8.01
	Ile	(α4^Thr261Ileu^)_2_(β2)_3_ (n = 3)	9.94 (9.58–10.31)	<0.0001	7.31
	Val	(α4^Thr261Val^)_2_(β2)_3_ (n = 3)	5.41 (5.27–5.56)	0.0010	3.98
	Ala	(α4^Thr261Ala^)_2_(β2)_3_ (n = 3)	1.08 (1.02–1.13)	0.9774	0.794
Intersubunit					
	β2^Asn215Val^	(α4)_2_(β2^Asn215Val^)_3_ (n = 3)	0.858 (0.816–0.899)	0.8471	0.631
		(α4^Thr261Ile^)_2_(β2^Asn215Val^)_3_ (n = 3)	3.19 (3.06–3.31)	0.1515	2.35
Intrasubunit					
		(α4^259Leu260 +Ile274Ala^)_2_(β2)_3_ (n = 3)	2.46 (2.37–2.55)	0.1823	1.81

Inspection of the recordings also reveals that channel opening episodes appear as bursts of one or more openings in quick succession, with the number of openings per burst increasing for the pathogenic variant compared to WT. To quantify the number of channel openings per burst, we plotted the fraction of bursts with greater than N channel reopenings, where N is an integer, against the number of reopenings per burst and fitted a single exponential to the data (Experimental procedures). Mean values of the fitted exponential decay constant reveal that the pathogenic variant reopens 2.6 times more per burst compared to WT (Table 2).

**Table 2:** Effect of α4 variants on mean number of channel reopenings per burst

Position of inserted residue	Residue	Receptor type (number of patches)	Mean reopenings per burst (95% CI)	*p* value for WT *versus* mutant	Fold change for mutant relative to WT
	WT	(α4)_2_(β2)_3_ (n = 3)	0.48 (0.47–0.50)	-	-
259–260	Leu	(α4^259Leu260^)_2_(β2)_3_ (n = 3)	1.26 (1.24–1.32)	0.0378	2.62
	Ile	(α4^259Ile260^)_2_(β2)_3_ (n = 3)	1.95 (1.83–2.07)	0.0129	4.06
	Val	(α4^259Val260^)_2_(β2)_3_ (n = 3)	1.02 (0.935–1.10)	0.0127	2.13
	Ala	(α4^259Ala260^)_2_(β2)_3_ (n = 3)	1.20 (1.05–1.37)	0.0223	2.5
	WT	(α4)_3_(β2)_2_ (n = 3)	0.64 (0.61–0.68)	-	-
	Leu	(α4^259Leu260^)_3_(β2)_2_ (n = 3)	1.23 (1.14–1.32)	0.0083	1.93
260–261	Leu	(α4^260Leu261^)_2_(β2)_3_ (n = 3)	2.31 (2.21–2.42)	0.0084	4.81
	Ile	(α4^260Ile261^)_2_(β2)_3_ (n = 3)	1.38 (1.29–1.48)	0.0204	2.87
	Ala	(α4^260Ala261^)_2_(β2)_3_ (n = 3)	0.86 (0.81–92)	0.0328	1.79
261–262	Val	(α4^261Val262^)_2_(β2)_3_ (n = 3)	1.48 (1.32–1.65)	0.0089	3.08
	Ala	(α4^261Ala262^)_2_(β2)_3_ (n = 3)	1.19 (1.11–1.26)	0.0307	2.48
262–263	Leu	(α4^262Leu263^)_2_(β2)_3_ (n = 3)	1.67 (1.46–1.89)	0.0062	3.47
	Ala	(α4^262Ala263^)_2_(β2)_3_ (n = 3)	1.16 (1.07–1.27)	0.0163	2.41
268–269	Ala	(α4^268Ala269^)_2_(β2)_3_ (n = 3)	0.28 (0.27–0.28)	0.2471	0.54
270–271	Ala	(α4^270Ala271^)_2_(β2)_3_ (n = 3)	0.29 (0.28–0.30)	0.2549	0.60
substituted residue in α4					
261	Leu	(α4^Thr261Leu^)_2_(β2)_3_ (n = 3)	1.02 (0.94–1.10)	0.0194	2.13
	Ile	(α4^Thr261Ileu^)_2_(β2)_3_ (n = 3)	1.01 (0.94–1.09)	0.0367	2.10
	Val	(α4^Thr261Val^)_2_(β2)_3_ (n = 3)	0.60 (0.55–0.61)	0.0929	1.25
	Ala	(α4^Thr261Ala^)_2_(β2)_3_ (n = 3)	0.45 (0.43–0.47)	0.5148	0.93
Intersubunit					
	β2^Asn215Val^	(α4)_2_(β2^Asn215Val^)_3_ (n = 3)	0.50 (0.48–0.53)	0.0862	1.04
		(α4^Thr261Ile^)_2_(β2^Asn215Val^)_3_ (n = 3)	0.83 (0.79–0.87)	0.0319	1.73
Intrasubunit					
	α4^259Leu260 +Ile274Ala^	(α4^259Leu260 +Ile274Ala^)_2_(β2)_3_(n = 3)	1.41 (1.33–1.50)	0.0226	2.94

To capture the changes in mean open time and channel reopening in a single parameter, we define the burst duration as the sum of the durations of channel openings in quick succession plus the intervening brief closings (Experimental procedures), which reflects the overall impact on channel gating. The resulting analysis reveals that the pathogenic variant increases the mean burst duration by 11-fold relative to WT for each stoichiometric form. As a check, we multiply the observed mean open time by the observed number of channel openings per burst, which gives a lower bound estimate of the mean burst duration. The observed and estimated mean burst durations are similar (Table 3); for example, for the low conductance stoichiometry, the observed mean burst durations are 1.7 and 19.5 ms for the WT and pathogenic variant, respectively, while the calculated values are 2.0 and 21.1 ms. Thus, the pathogenic variant promotes gain of function by stabilizing the open state, increasing channel reopening, and increasing the burst duration. All three functional readouts indicate that for each stoichiometric form the pathogenic variant enhances gating of the receptor channel.

**Table 3:** Effect of α4 insertion mutations on mean burst duration

Position of inserted residue	Residue	Receptor type (number of patches)	Mean burst duration (ms) (95% CI)	*p* value for WT *versus* mutant	Fold change for mutant relative to WT	Calculated burst duration
	WT	(α4)_2_(β2)_3_ (n = 3)	1.66 (1.61–1.71)	-	-	2.02
259–260	Leu	(α4^259Leu260^)_2_(β2)_3_ (n = 3)	19.1 (17.8–20.4)	<0.0001	11.5	21.6
	Ile	(α4^259Ile260^)_2_(β2)_3_ (n = 3)	12.9 (11.9–13.8)	0.0010	7.77	15.1
	Val	(α4^259Val260^)_2_(β2)_3_ (n = 3)	13.1 (12.3–13.9)	0.0008	7.89	14.1
	Ala	(α4^259Ala260^)_2_(β2)_3_ (n = 3)	17.2 (12.5–21.9)	<0.0001	10.36	20.7
	WT	(α4)_3_(β2)_2_ (n = 3)	1.50 (1.41–1.58)	-	-	1.93
	Leu	(α4^259Leu260^)_3_(β2)_2_ (n = 3)	16.6 (14.9–18.3)	<0.0001	11.1	17.6
260–261	Leu	(α4^260Leu261^)_2_(β2)_3_ (n = 3)	4.66 (4.37–4.95)	0.4790	2.81	5.46
	Ile	(α4^260Ile261^)_2_(β2)_3_ (n = 3)	9.34 (8.69–9.99)	0.0180	5.62	10.9
	Ala	(α4^260Ala261^)_2_(β2)_3_ (n = 3)	16.2 (14.2–18.1)	<0.0001	9.73	20.5
261–262	Val	(α4^261Val262^)_2_(β2)_3_ (n = 3)	4.01 (3.65–4.37)	0.0211	2.41	5.11
	Ala	(α4^261Ala262^)_2_(β2)_3_ (n = 3)	4.05 (3.87–4.23)	0.0213	2.44	4.66
262–263	Leu	(α4^262Leu263^)_2_(β2)_3_ (n = 3)	4.32 (3.88–4.76)	0.0219	2.60	3.92
	Ala	(α4^262Ala263^)_2_(β2)_3_ (n = 3)	4.91 (4.63–5.19)	0.0033	2.96	5.89
268–269	Ala	(α4^268Ala269^)_2_(β2)_3_ (n = 3)	0.64 (0.58–0.69)	<0.0001	0.389	0.78
270–271	Ala	(α4^270Ala271^)_2_(β2)_3_ (n = 3)	0.96 (0.92–1.01)	<0.0001	0.578	1.23
substituted residue in α4						
261	Leu	(α4^Thr261Leu^)_2_(β2)_3_ (n = 3)	21.4 (19.9–22.7)	<0.0001	12.9	22.1
	Ile	(α4^Thr261Ileu^)_2_(β2)_3_ (n = 3)	16.0 (15.1–16.9)	<0.0001	9.64	19.9
	Val	(α4^Thr261Val^)_2_(β2)_3_ (n = 3)	6.97 (6.74–7.20)	0.0235	4.21	8.66
	Ala	(α4^Thr261Ala^)_2_(β2)_3_ (n = 3)	1.26 (1.20–1.34)	0.9963	0.76	1.56
Intermolecular						
	β2^Asn215Val^	(α4)_2_(β2^Asn215Val^)_3_ (n = 3)	1.08 (1.02–1.13)	0.9029	0.65	0.948
		(α4^Thr261Ile^)_2_(β2^Asn215Val^)_3_ (n = 3)	5.40 (5.14–5.65)	0.0297	3.25	4.29
Intramolecular						
		(α4^259Leu260 +Ile274Ala^)_2_(β2)_3_(n = 3)	4.88 (4.64–5.12)	0.1428	2.94	4.36

**Figure 3:**
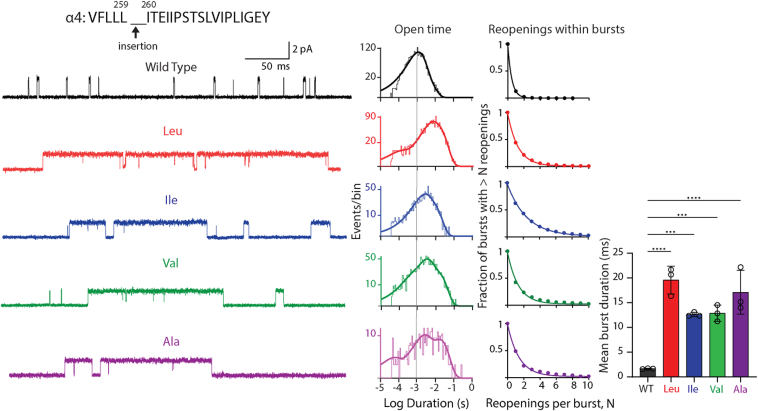
**Impact of varying the size of the inserted residue between positions 259 and 260 of the α4 subunit.** Exemplar traces are displayed for WT and each inserted residue at 5 kHz bandwidth. Alongside each trace are the corresponding open time histogram fitted by the sum of exponentials and a plot of channel reopening probability fitted by a single exponential. *Vertical line* through the open time histograms indicates the mean open duration of the WT receptor. *Bar graph* compares the mean duration of all bursts for WT and receptors with residue insertions color coded to the corresponding trace and histogram. The recordings were obtained using the cell-attached patch configuration in the presence of 1 μM ACh at a membrane potential of −70 mV. N = 3 recordings per mutant for burst duration analysis. Data are expressed as mean ± SD and analyzed by one-way ANOVA. Differences between WT and mutant are indicated by ∗∗∗∗*p* < 0.0001 and ∗∗∗*p* < 0.001. See Tables 2 and 3 for reopening decay constants, mean burst durations, and *p* values.

Having established that the pathogenic variant enhances receptor channel gating, we sought to understand structural bases for this enhancement. A residue insertion has two possible effects: altered inter-residue interactions local to the insertion and propagated effects due to residue shift along the protein chain. To assess changes in local inter-residue interactions, we altered the size of the side chain of the inserted residue and recorded ACh-elicited single-channel currents. Hereafter, our studies focus on the low conductance (α4)_2_(β2)_3_ stoichiometric form as it showed relatively greater expression levels and thus an increased fraction of membrane patches with sufficient channel opening activity. Recordings from receptors with the hydrophobic insertions Ile, Val, and Ala between positions α4-259 and α4-260 show prolonged channel openings, with the open duration histograms and mean duration of all openings similar to those of the pathogenic variant (Fig. 3; Table 1). For each hydrophobic insertion, the mean number of channel reopenings per burst is greater relative to WT but similar to that for the pathogenic variant (Table 2). The combined changes in open time and reopening per burst, captured by the mean burst duration, increase relative to WT but are similar to the pathogenic variant (Table 3). Thus, the effect of the pathogenic variant is independent of the size of the inserted residue. The observation of size independence suggests residue shift along the protein chain contributes to enhanced channel gating, although contributions by altered inter-residue interactions local to the insertion are also possible.

**Figure 4:**
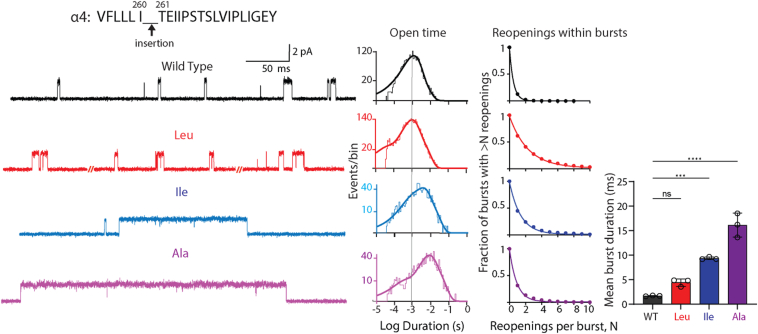
**Impact of residue insertions of differing size between positions 260 and 261 of the α4 subunit.** Exemplar traces are displayed for WT and each inserted residue at 5 kHz bandwidth. Alongside each trace are the corresponding open time histogram fitted by the sum of exponentials and a plot of channel reopening probability fitted by a single exponential. *Vertical line* through the open time histograms indicates the mean open duration of the WT receptor. *Bar graphs* compare the mean duration of all bursts for WT and receptors with residue insertions color coded to the corresponding trace and histogram. The recordings were obtained using the cell-attached patch configuration in the presence of 1 μM ACh at a membrane potential of −70 mV. N = 3 recordings per mutant for burst duration analysis. Data are expressed as mean ± SD and analyzed by one-way ANOVA. Differences between WT and mutant are indicated by ∗∗∗∗*p* < 0.0001 and ∗∗∗*p* < 0.001. See Tables 2 and 3 for reopening decay constants, mean burst durations, and *p* values. The data shown for WT are from Figure 3 for ease of comparison.

To explore contributions due to residue shift, we inserted a range of hydrophobic residues one position further down the protein chain. Recordings from the insertions α4^260Insert261^ reveal a systematic increase in the duration of individual channel openings as the size of the inserted side chain decreases from Leu to Ala (Fig. 4). The increases in open duration are accompanied by shift of the open time histogram toward longer durations and increases of the mean duration of all openings (Table 1). Intriguingly, channel reopening shows the opposite trend, with the mean number of reopenings per burst decreasing as the size of the inserted side chain decreases (Table 2). The net effect on channel gating, measured by mean burst duration, shows a progressive increase as the size of the side chain decreases (Table 3). These results further suggest the effects of the original α4^259Leu260^ pathogenic insertion are due to residue shift, but they also reveal contributions of size and/or hydrophobicity by the adjacent residue in the C-terminal direction.

**Figure 5:**
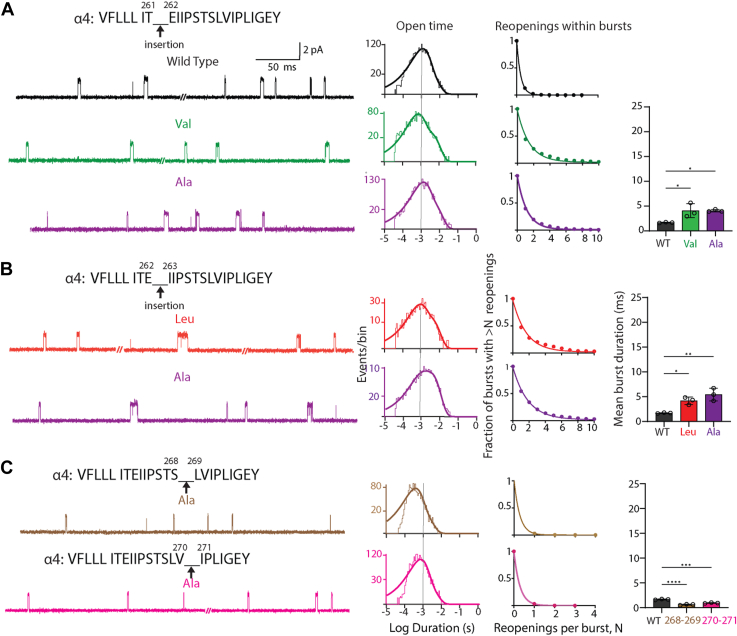
**Impact of additional residue insertions in the C-terminal direction from the pathogenic insertion.***A*, insertions between residues 261 and 262 within M2. *B*, insertions between residues 262 and 263 within M2. *C*, insertions between residues 268 and 269 and between 270 and 271 within the M2-M3 linker. Alongside each trace are the corresponding open time histogram fitted by the sum of exponentials and a plot of channel reopening probability fitted by a single exponential. *Vertical line* through the open time histograms indicates the mean open duration of the WT receptor. *Bar graphs* compare the mean duration of all bursts for WT and receptors with residue insertions color coded to the corresponding trace and histogram. The recordings were obtained using the cell-attached patch configuration in the presence of 1 μM ACh at a membrane potential of −70 mV and displayed at 5 kHz bandwidth. N = 3 recordings per mutant for burst duration analysis. Data are expressed as mean ± SD and analyzed by one-way ANOVA. Differences between WT and mutant are indicated by ∗∗∗∗*p* < 0.0001, ∗∗*p* < 0.0034, and ∗*p* < 0.023. See Tables 2 and 3 for reopening decay constants, mean burst durations, and *p* values. The data shown for WT in *panel A* are from Figure 3 for ease of comparison.

To further explore effects due to residue shift, we inserted hydrophobic residues of different sizes two and three positions in the C-terminal direction with respect to the pathogenic insertion (α4^261Insert262^ and α4^262Insert263^). Like the pathogenic insertion, these insertions are located within the M2 α-helix. For both insertions, the recordings show individual channel openings similar to WT and open duration histograms with small shifts to longer durations (Fig. 5, *A* and *B*). Quantitative analysis reveals that relative to WT the mean durations of all openings are prolonged approximately 2-fold, and the mean number of channel reopenings per burst either do not change or increase up to 2-fold (Tables 1 and 2). The changes in open duration and channel reopening produce modest increases in mean burst duration that are much smaller than that of the pathogenic insertion α4^259Leu260^ (Table 3). Thus, residue insertions several positions beyond the pathogenic insertion have a smaller impact on channel gating than the pathogenic insertion.

To further explore effects due to residue shift, we inserted an Ala residue at two positions within the linker spanning the M2 and M3 α-helices. For both insertions, the recordings show somewhat briefer channel openings than WT and open duration histograms with small shifts to shorter durations (Fig. 5*C*). Quantitative analysis reveals that the mean durations of all openings are 2-fold briefer or less than WT, with negligible reopenings per burst, resulting in essentially identical values for mean open and burst durations (Tables 1 and 2). Thus, residue insertions within the M2-M3 linker modestly suppress channel gating. The overall results of residue insertions show that structures that contribute to enhanced channel gating are confined to approximately one half turn of the M2 α-helix.

**Figure 6:**
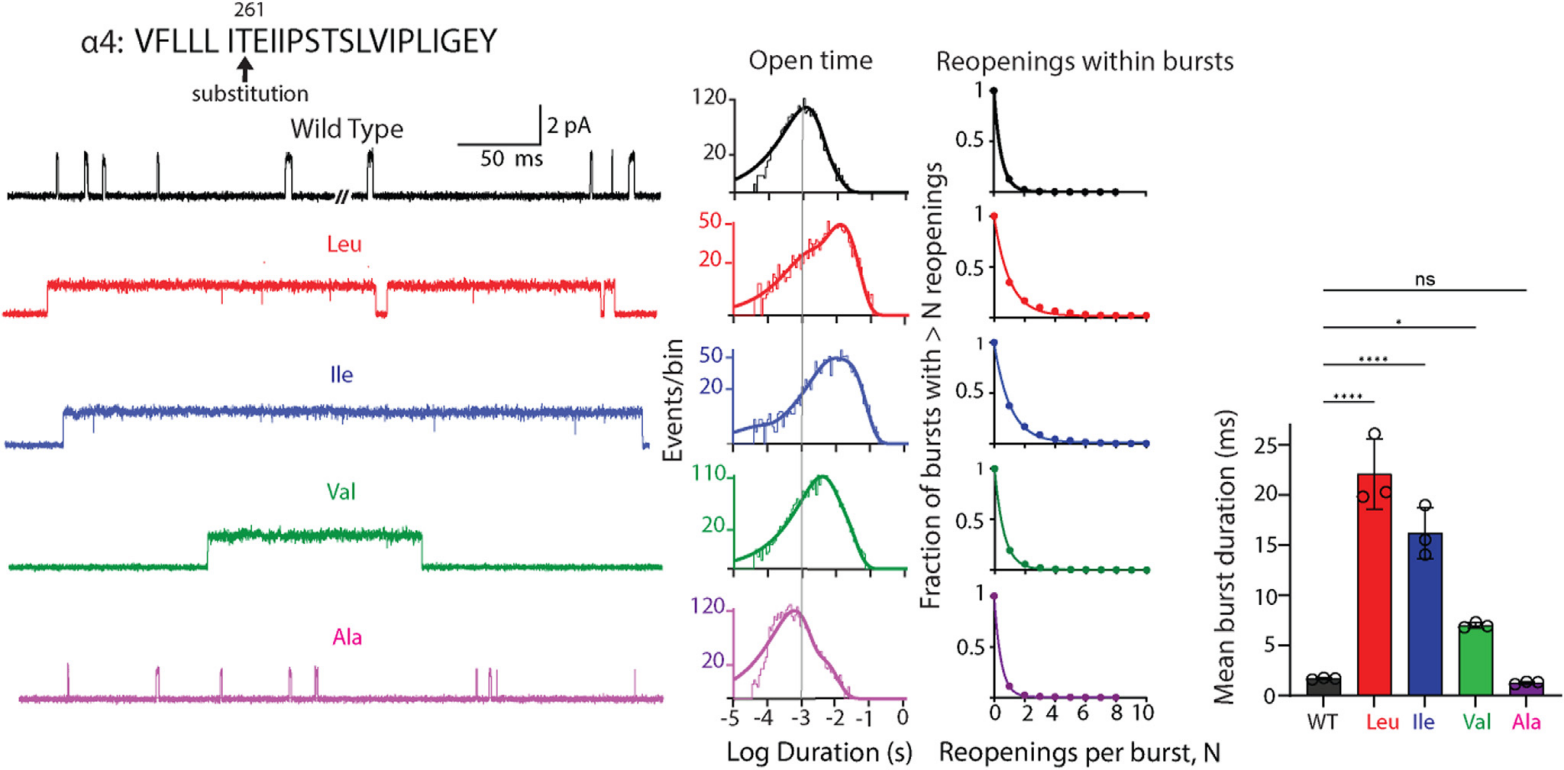
**Impact of substituting hydrophobic residues of varying size for α4-Thr261.** Exemplar traces are displayed for WT and the indicated substituted residue at 5 kHz bandwidth. Alongside each trace are the corresponding open time histogram fitted by the sum of exponentials and a plot of channel reopening probability fitted by a single exponential. *Vertical line* through the open time histograms indicates the mean open duration of the WT receptor. *Bar graphs* compare the mean duration of all bursts for WT and receptors with residue insertions color coded to the corresponding trace and histogram. The recordings were obtained using the cell-attached patch configuration in the presence of 1 μM ACh at a membrane potential of −70 mV. N = 3 recordings per mutant for burst duration analysis. Data are expressed as mean ± SD and analyzed by one-way ANOVA. Differences between WT and mutant are indicated by ∗∗∗∗*p* < 0.0001 and ∗*p* < 0.024. See Tables 2 and 3 for reopening decay constants, mean burst durations, and *p* values. The data shown for WT are from Figure 3 for ease of comparison.

To identify inter-residue interactions within the region of M2 encompassing the pathogenic insertion, we considered α4-Thr261 to be a strong candidate because residue shift due to the pathogenic insertion would place an Ile residue at the position originally occupied by α4-Thr261, which in the native structure makes polar interactions with the adjacent β2 subunit. Thus, we substituted hydrophobic residues of varying sizes for α4-Thr261 and recorded ACh-elicited single-channel currents. The recordings reveal that the largest substitutions, Leu and Ile, markedly prolong channel opening relative to WT, whereas the smaller Val prolongs less, and the smaller Ala produces slightly briefer channel openings (Fig. 6). The mean durations of all openings and the open duration histograms exhibit a similar pattern of progressive change from long to brief as residue size decreases. The mean number of channel reopenings per burst increases for Leu and Ile substitutions and progressively decreases for the Val and Ala substitutions. The dual changes in mean open time and channel reopening, measured by mean burst duration, show a strikingly linear relationship between size of the hydrophobic side chain and burst duration. Thus, steric and/or hydrophobic interactions at position α4-261 are pivotal in tuning channel gating.

**Figure 7:**
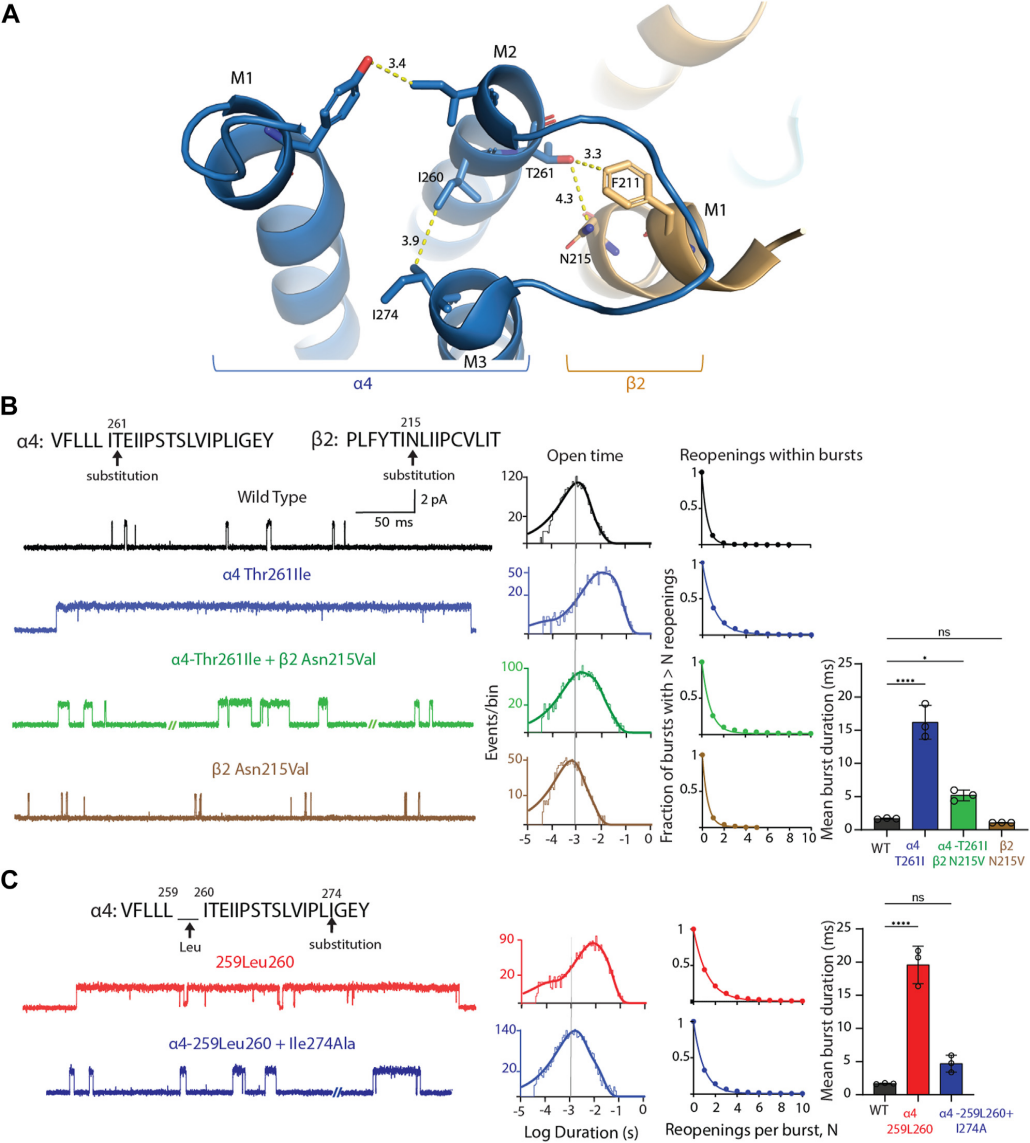
**Functional rescue reveals pairwise intersubunit and intrasubunit interactions that contribute to channel gating.***A*, *close up view* of the (α4)_2_(β2)_3_ nAChR (PDB: 6CNJ) from the extracellular side approximately normal to the plane of the membrane showing the α4 subunit in *blue* and β2 subunit in *beige* in *cartoon representation*. Key residues are highlighted in *stick representation* with interresidue distances in Angstroms indicated; for the T261 to N215 distance the *dotted line* spans from the hydroxyl oxygen atom to the amide nitrogen atom. *B* and *C*, local sequences encompassing residue substitutions or insertion plus substitution, respectively. For each panel, exemplar traces are displayed for WT and receptors with the indicated residue substitutions/insertions at 5 kHz bandwidth. Alongside each trace are the corresponding open time histogram fitted by the sum of exponentials and a plot of channel reopening probability fitted by a single exponential. *Vertical line* through the open time histograms indicates the mean open duration of the WT receptor. *Bar graphs* compare the mean duration of all bursts for WT and receptors with residue substitutions/insertions color coded to the corresponding trace and histogram. The recordings were obtained using the cell-attached patch configuration in the presence of 1 μM ACh at a membrane potential of −70 mV. N = 3 recordings per mutant for burst duration analysis. Data are expressed as mean ± SD and analyzed by one-way ANOVA. Differences between wild type and mutant are indicated by ∗∗∗∗*p* < 0.0001, ∗*p* < 0.03, and *p* > 0.05. See Tables 2 and 3 for reopening decay constants, mean burst durations, and *p* values. The data shown for WT in *panel B* and pathogenic variant in *panel C* are from Figure 3 for ease of comparison.

The relationship between hydrophobic side chain size and burst duration observed for substitutions at α4-Thr261 suggests a change in interresidue interactions. Inspection of the structure of the α4β2 receptor shows that α4-Thr261 closely juxtaposes Asn215 from the M1 α-helix of the adjacent β2 subunit (Fig. 7*A*). Thus, we hypothesized that increased burst duration arises from increasingly hydrophobic substitutions at position 261 of the α4 subunit that interact unfavorably with the hydrophilic residue Asn215 of the β2 subunit. To test this hypothesis, we combined the α4-Thr261Ile substitution with the hydrophobic substitution β2-Asn215Val and recorded ACh-elicited single-channel currents. The expectation is that a hydrophobic–hydrophobic interaction between the substituted Ile and Val residues would be more favorable than a hydrophobic–hydrophilic interaction between Ile and Asn. In accord with this expectation, the recordings reveal markedly briefer channel openings for the double mutant than the single α4-Thr261Ile mutant, with the single β2-Asn215Val mutant showing little change relative to WT (Fig. 7*B*). In further agreement, the double mutant shows a marked shift of the open time histogram toward briefer durations, while the single β2-Asn215Val mutant shows a modest shift to brief durations relative to WT; the changes in the mean duration of all openings parallels the changes in the open duration histograms (Table 1). Analyses of channel reopening and burst duration show that the double mutant approaches the corresponding measures for the WT receptor, while the single β2-Asn215Val mutant is essentially unchanged compared to WT (Tables 2 and 3). These results provide evidence for an intersubunit interaction between Thr261 of the α4 subunit and Asn215 of the β2 subunit that contributes to channel gating.

Further inspection of the structure of the α4β2 receptor suggests a change in intrasubunit interactions local to the pathogenic insertion α4^259Leu260^. In particular, the inserted Leu residue would take the place of the adjacent Ile residue at position 260, which in the WT receptor closely approaches α4-Ile274 from the M3 α-helix (Fig. 7*A*). The side chain of the inserted Leu residue contains an additional terminal methyl group compared to the original Ile side chain at position 260, suggesting a steric clash between the inserted Leu residue and α4-Ile274 in M3. To test this idea, we substituted a smaller Ala residue for α4-Ile274 while retaining the pathogenic Leu insertion α4^259Leu260^. Recordings of ACh-elicited single-channel currents reveal markedly briefer channel openings for the double mutant receptor compared to the pathogenic insertion α4^259Leu260^ alone (Fig. 7*C*). Accordingly, the double mutant shows a marked shift of the open time histogram toward briefer durations and a decrease of the mean duration of all openings compared to the pathogenic insertion alone (Table 1). Analyses of channel reopening reveals no change for the double mutant relative to the pathogenic insertion. However, owing to the decrease in open duration, the burst duration for the double mutant is markedly reduced compared to the pathogenic insertion and approaches that of the WT receptor (Table 3). These results provide evidence that an intrasubunit interaction between the pathogenic Leu insertion in M2 and α4-Ile274 in M3 contributes to channel gating.

**Figure 8:**
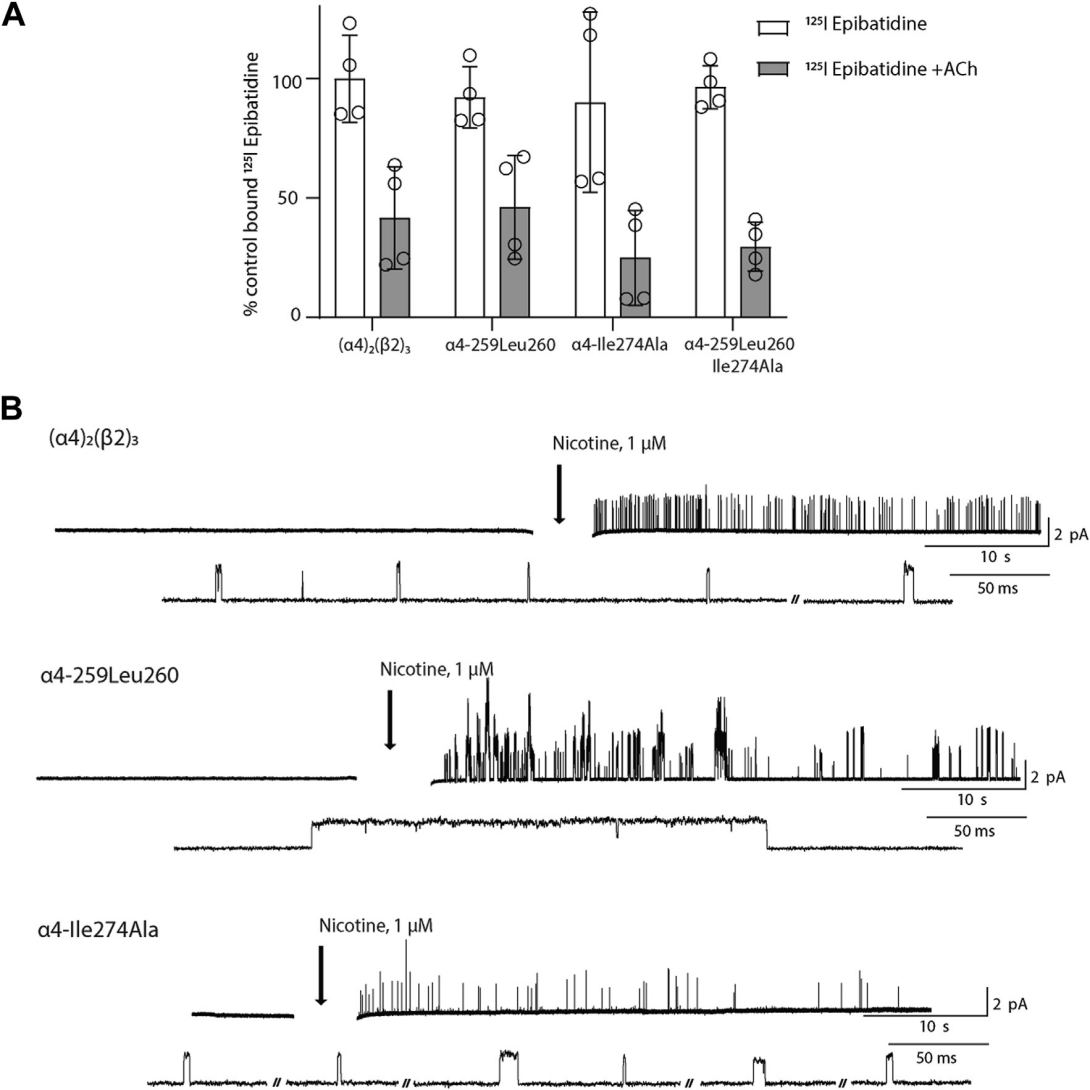
**Expression and agonist-dependent single-channel activity of single and pairwise mutant receptors.***A*, radio-labeled epibatidine binding to intact Bosc 23 cells–expressing WT and mutant receptors was measured as described in Experimental procedures. Results from two independent transfections, each in duplicate, are shown, with control binding defined as that measured for the WT receptor in the presence of epibatidine alone. Net cell surface binding is the difference between binding in the presence of epibatidine alone (*open bars*) and that in the presence of ACh (*gray bars*); binding in the presence of ACh corresponds to intracellular binding sites. *B*, single-channel recordings from the same of patch of cell membrane before and after addition of nicotine (final concentration 100 nM) to the bath solution for the indicated receptors. Traces are representative of three experiments per receptor. The electrical artifact during nicotine addition is removed. The cell attached patch configuration was maintained throughout with a membrane potential of −70 mV and display bandwidth of 2 kHz.

A complete interpretation of the results in Figure 7*C* requires control recordings from the single mutant α4Ile274Ala, however despite many attempts, single-channel currents were not observed using the cell-attached patch clamp configuration. Measurements of radio-labeled epibatidine binding to intact cells show that expression of the α4-Ile274Ala mutant is similar to that of WT and the double mutant receptors (Fig. 8*A*), as shown by either total epibatidine binding or cell-surface binding determined from the difference between total binding and binding in the presence of ACh. Notably, binding in the presence of ACh, which represents binding to intracellular receptors, is a significant fraction of the total binding for WT and mutant receptors. The absence of detectable channel openings together with robust epibatidine binding suggest α4-Ile274Ala either reduces the probability a channel will open or increases desensitization such that channel opening activity disappears in the time required to form a cell-attached patch. Regardless of whether α4-Ile274Ala impacts channel opening or desensitization, this result is interesting because this mutant rescues aberrant channel gating by the pathogenic insertion.

To test for possible increased desensitization by α4-Ile274Ala, we devised an experiment to monitor single-channel activity from the same patch of membrane before and after agonist application. To do this, we first established a cell-attached patch without agonist in the patch pipette, and after a control period in the absence of agonist, the membrane-permeable agonist nicotine was added to the external solution. The recordings show that WT receptors are inactive in the absence of nicotine, but they activate following nicotine addition, as expected (Fig. 8*B*). Similarly, the pathogenic mutant receptor shows the same profile of electrical silence in the absence of nicotine and robust single-channel activity following nicotine addition. The observation of electrical silence in the absence of agonist shows that pathogenic mutant receptors do not open spontaneously, in contrast to previously described mutations in M2 that increase spontaneous channel opening (2, 13, 14, 15). Finally, for the α4-Ile274Ala mutant, following electrical silence in the absence of nicotine, the addition of nicotine elicits relatively sparse channel openings, which decline in frequency as the recording progresses. Thus, although the α4-Ile274Ala mutant shows ample cell-surface expression and detectable channel opening on initial exposure to agonist, in the cell-attached patch configuration channel opening is not observed likely due to reduced channel opening probability, increased desensitization, or a combination of the two. Notably these suppressive effects by the α4-Ile274Ala mutant are countered by combining it with the pathogenic insertion.

**Figure 9:**
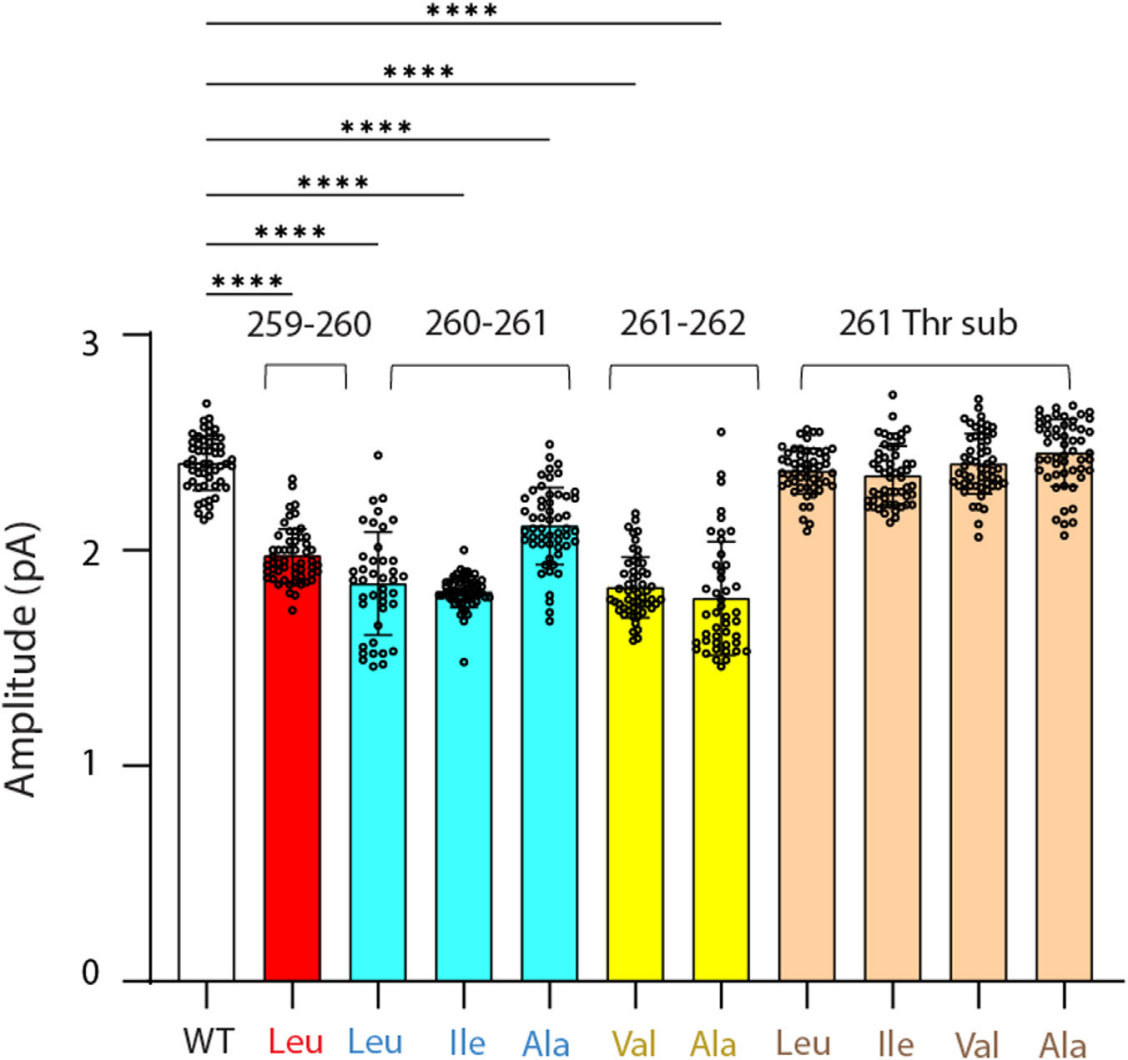
**Residue insertions reduce whereas residue substitutions do not affect the unitary current amplitude.** For each WT or mutant receptor, the current amplitude is the mean value from three independent patches at a membrane potential of −70 mV with error bars showing the SD. For a specified mutant, each data point represents the current amplitude from a 50 ms sweep of the raw current trace, with the total number of sweeps N ranging from 45 to 65 per mutant. Data are expressed as mean ± SD and analyzed by one-way ANOVA ∗∗∗∗*p* < 0.0001. For the 261 Thr substitutions, *p* > 0.05. Results are summarized in Table S1.

So far the focus has been on changes in the kinetics of channel opening and closing by the pathogenic insertion. However, in the course of these studies, we noticed a modest reduction in the unitary current amplitude. This reduction may be expected due to the pathogenic insertion causing a residue shift. As a result, the pore-facing Glu262, which contributes to the external ring of charge that contributes to ion conduction (16), would be expected to rotate out of the pore. Analyses of current amplitude reveals an approximately 20% reduction in mean current amplitude for residue insertions between the following positions: 259 to 260, 260 to 261, and 261 to 262 (Fig. 9; Table S1). By contrast, mean current amplitudes for residue substitutions for Thr261, which do not cause a residue shift, are indistinguishable from that of WT. These findings suggest that the insertions within M2 studied herein produce a residue shift along the protein chain such that Glu262 is rotated away from the pore.

## Discussion

Naturally occurring pathogenic mutations can be recognized because they alter a functionally crucial part of a protein’s structure. As such they represent starting points to delineate structure-function relationships in physiologically pivotal proteins. Here, we genetically reconstitute a pathogenic single residue Leu insertion in the α4β2 nAChR associated with sleep-related hyperkinetic epilepsy and use single-channel patch clamp electrophysiology to identify altered steps within the process of receptor activation by agonist. The results reveal that the pathogenic insertion selectively stabilizes the open channel state, enhancing the terminal channel gating step. We also combine single-channel recording with site-directed mutagenesis to distinguish whether residue shift by the insertion or changes in local inter-residue interactions underpin the enhancement of channel gating. The results reveal that both types of structural changes impact channel gating, but that these changes are confined to a localized region near the inserted residue. Within this region we identify changes in both intra- and inter-subunit interactions that underpin enhanced channel gating, and show that the enhancement of channel gating may be countered by changes in the size or hydrophobicity of contact residues. The overall results reveal a novel inter-helical region in which hydrophobic and/or steric interactions tune the stability of the open channel state.

Patch clamp recording of single ion channel currents allows one to directly detect transitions between conducting open and non-conducting closed states and relate these transitions to elementary steps in the process of receptor activation by agonist. The primary effect of the pathogenic insertion is to markedly increase the mean open channel lifetime, indicating a slowing of the rate at which the open channel returns to the closed state. A secondary effect is to increase channel reopening or the probability a channel opens multiple times per activation episode; reopening is determined by the rate at which the closed state following a channel opening transitions back to the open state as opposed to transitioning to the long-lived resting closed state. A third parameter, the mean burst duration, combines open channel lifetime and channel reopening into a single quantity, representing the impact on receptor channel gating. Burst duration has the further advantage that it does not depend on the recording bandwidth of the patch clamp; reducing or increasing the bandwidth will alter both the apparent channel open time and apparent channel reopening, but these two measures change in a reciprocal manner that does not alter the burst duration. A final parameter, channel opening in the absence of agonist, is not observed for either the WT or pathogenic insertion receptors. Notably, however, numerous other gain-of-function mutations within M2 cause spontaneous channel opening (2, 13, 14, 15), thus the pathogenic insertion is novel in remaining electrically silent in the absence of agonist.

One of the first pathogenic mutations of an nAChR was discovered nearly 30 years ago, a gain-of-function mutation in the muscle nAChR that prolonged open channel lifetime and caused spontaneous channel opening (2). Since then many pathogenic mutations, both loss and gain of function, have been identified in both muscle and neuronal nAChRs (4, 6). Most gain-of-function mutations localize to the M2 transmembrane domain, which is one of four transmembrane domains in each of the five receptor subunits. The five M2 domains congregate to form the central ion conductance pathway and are flanked by the other transmembrane domains by the same and adjacent subunits. With the advent of high-resolution nAChR structures, pathogenic gain-of-function mutations can be accurately mapped onto the receptor structure, which reveals that the side chains of most M2 mutations project either into the ion channel or toward M2 of the adjacent subunit (Table S2). However, the pathogenic insertion studied herein is structurally novel in that its side chain projects away from the ion channel, interacting with the M1 and M3 α-helices of the same subunit.

A residue insertion is expected to shift flanking residues along the protein chain and alter inter-residue interactions. In principle, the shift of flanking residues could be either in the N- or C-terminal direction. However, the pathogenic insertion is located within the last turn of the M2 α-helix such that a residue shift in the C-terminal direction seems more likely. Although a C-terminal shift is our working hypothesis, an N-terminal shift remains possible in the absence of direct structural information. In support of a residue shift, the pathogenic insertion reduces the unitary current amplitude ∼20%, consistent with rotation of α4-Glu262 away from the pore and reducing its ability to stabilize permeant cations. Furthermore, in the case of a C-terminal shift, a Leu residue inserted between positions 259 and 260 of the α4 subunit would take the place of the Ile260 residue, which in the native structure contacts Ile274 from M3 of the same subunit, potentially altering interactions between the M2 and M3 α-helices (Fig. 7*A*). The displaced Ile260 residue would then take the place of the Thr261 residue, which in the native structure contacts Asn215 and Phe211 residues from M1 of the neighboring β2 subunit, creating a mismatch of both size and hydrophobicity. Finally, the displaced Thr261 residue would take the place of the pore-facing Glu262 residue, which would take the place of the Ile263 residue that in the native structure contacts the Tyr213 residue from M1 of the same subunit, potentially creating a novel Glu-Tyr hydrogen bond. Thus, the pathogenic Leu insertion is expected to alter three interhelical contacts, two within the same α4 subunit and one between α4 and β2 subunits.

We find that the effects of structural perturbations due to the pathogenic insertion are confined to about one half turn of the M2 α-helix. Residue insertions in the α4 subunit at 259 to 260 and 260 to 261 within M2 markedly impact the burst duration, whereas insertions at 261 to 262 and 262 to 263 within M2 and 268 to 269 and 270 to 271 within the M2-M3 linker have modest or no effect on burst duration. Thus, the structural region that most strongly impacts burst duration spans about 200° of a turn of the M2 α-helix of the α4 subunit, suggesting changes in local interactions with the surrounding α-helices.

Our studies provide evidence that two of the three interhelical interactions evident in the high-resolution structure of the α4β2 nAChR impact burst duration. The first of these interhelical interactions is demonstrated by combining the pathogenic Leu insertion with substitution of a predicted contact residue within the M3 α-helix of the same subunit, α4-Ile274Ala; the combined mutant restores burst duration to approach that of the WT receptor, revealing an intrasubunit interaction contributing to channel gating. Secondly, given a C-terminal residue shift, the pathogenic insertion would advance Ile260 into the position originally occupied by Thr261. We find that while the substitution α4-Thr261Ile markedly prolongs burst duration, combining it with the substitution β2-Asn215Val counteracts the increase in burst duration, suggesting that the resulting hydrophobic–hydrophobic interaction relieves the hydrophobic–hydrophilic mismatch due to α4-Thr261Ile alone. Thus, within the local region of M2 impacted by the pathogenic mutation, two interhelical contacts, one intrasubunit, and another intersubunit impact channel gating.

**Figure 10:**
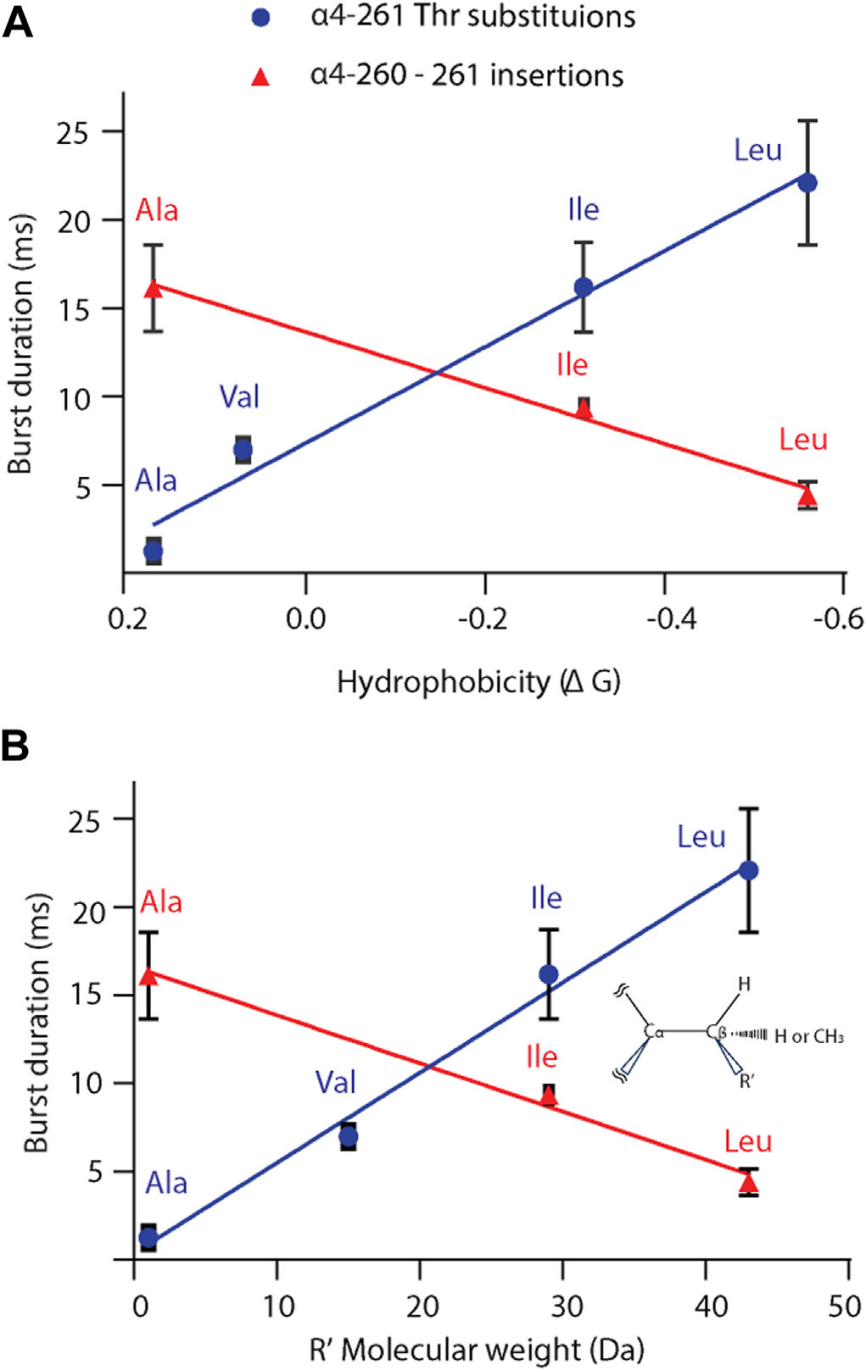
**Contributions of hydrophobicity and molecular weight to channel gating for residue substitutions at α4-Thr261 (*blue*) and insertions at α4-260 to 261 (*red*).***A*, plot of mean burst duration against hydrophobicity of the substituted or inserted residue (17). *B*, plot of mean burst duration against the molecular weight of the R′ moiety attached to the β carbon of the substituted or inserted residue. *Lines* are least squares fits. N = 3 recordings for each mutant. Data are expressed as mean ± SD.

To further test an intersubunit interaction between α4-Thr261 and the neighboring β2-subunit, we substituted various hydrophobic residues for α4-Thr261 and observed a systematic increase in burst duration as hydrophobicity of the substituted residue increased. A plot of burst duration against residue hydrophobicity reveals a strikingly linear relationship (Fig. 10); here residue hydrophobicity was measured by the free energy of transfer of a model peptide containing a test residue from a phospholipid bilayer to water (17). We also noted that a plot of the molecular weight of the moeity attached to the β-carbon of the substituted residue against burst duration also shows a linear relationship. Thus, increases in both hydrophobicity and size of the β-carbon moiety of the residue substituted for α4-Thr261 correlate with increases in burst duration.

We also observe a linear relationship between burst duration and hydrophobicity/molecular weight for residue insertions between α4 positions 260 and 261. However, for these insertions, increases in hydrophobicity/molecular weight correlate with decreases in burst duration, opposite to the trend observed for substitutions at α4-Thr261. This result is paradoxical because the inserted residue is expected to occupy the position of the native α4-Thr261. However, a residue insertion also shifts adjacent residues along the protein chain, creating new inter-residue interactions. In particular, in the native structure α4-Glu262 contributes to the outer ring of charge that contributes to ion conduction, but a C-terminal shift of the protein chain would rotate it out of the pore by 100°, where it would replace α4-Ile263 that contacts α4-Tyr213 from M1 of the same subunit, potentially creating a Glu-Tyr hydrogen bond. Evidence that Glu262 rotates out of the pore is our observation that the unitary current amplitude decreases for residue insertions but not substitutions. The potential Glu-Tyr hydrogen bond could alter the tilt of M2 such that changes in hydrophobicity/size of the inserted residue have the opposite effect compared to residue substitution alone. Although this interpretation may be speculative, the results of the 260 to 261 residue insertions show that in addition to changes in local inter-residue interactions, changes due to residue shift also impact burst duration.

The linear relationships described herein are reminiscent of a previous study of a pathogenic loss-of-function missense mutation in M3 of the muscle nAChR α-subunit, α1-V285I. In that study, residue substitutions for α1-V285 altered channel gating efficiency in proportion to the size and volume of the moiety attached to the β-carbon of the substituting residue (18). This dependence on the size of the β-carbon moeity, as opposed to the entire side chain, indicated a stereochemical contribution to channel gating, as might be expected from an asymmetric arrangement of neighboring structures. Although not noted in the previous work, the impact on channel gating also correlated with hydrophobicity of the substituting residue. Thus, the observations in both the present and previous study show that channel gating is modulated by size, hydrophobicity, and stereochemical features of the side chains mediating interactions between transmembrane domains of the same and adjacent subunits.

## Experimental procedures

### Drugs

Acetylcholine chloride and nicotine were purchased from Sigma-Aldrich.

### Receptor expression and mutagenesis

Plasmids containing complementary DNAs (cDNAs) encoding α4 and β2 subunits and the chaperones NACHO and 14-3-3η were cloned individually into the mammalian expression vector pCI (Promega) or pRBG4, as previously described (19). Mutations were introduced by identifying a pair of unique restriction sites on either side of the desired mutation and cloning a synthetic double-stranded oligonucleotide (Integrated DNA Technologies), harboring the mutation between the unique sites using standard molecular biological methods. The presence of the intended mutations and the absence of unwanted mutations were confirmed by sequencing. Bosc 23 cells, an HEK293-derived cell line (CLS Cat# 300192/p777_HEK293, RRID:CVCL_0045) (20), were used to express WT and mutant receptors. Cells were seeded in 35 mm tissue culture plates and maintained in Dulbecco's modified Eagle's medium (Gibco) with 10% fetal bovine serum until they reached 50% confluency, after which they were transfected with cDNAs encoding either WT or mutant receptors using calcium phosphate precipitation. To identify transfected cells for patch clamp recording, a cDNA encoding GFP was included in all transfections. 14-3-3η and NACHO were used to bias expression toward either the (α4)_3_(2)_2_ or (α4)_2_(β2)_3_ stoichiometric forms (11), respectively, and ratios of subunits in the transfection were also biased as follows: 1:1:0.4 α4:β2:NACHO for (α4)_2_(β2)_3_ and 10:1:10 4:b2:1433η for (α4)_3_(β2)_2_. After a 7 h transfection period followed by media exchange, patch-clamp recordings were made 48 to 72 h afterward.

### Single-channel patch clamp recording

Single-channel currents were recorded in the cell-attached patch configuration at a membrane potential of −70 mV and temperature of 22 °C. Pipette and extracellular bath solutions contained (mM) 5.4 NaCl, 142 KCl, 1.8 CaCl_2_, 1.7 MgCl_2_, 10 Hepes, adjusted to pH 7.4 with NaOH. At the time of recording, growth medium was removed from the 35 mm tissue culture plate containing the cells and 0.9 ml of extracellular bath solution was added. Concentrated 1 mM stock solutions of ACh (Sigma-Aldrich) were stored at −80 °C and diluted to 1 μM in pipette solution on the day of each experiment. Patch pipettes were fabricated from glass capillary tubes (7052, King Precision Glass), coated with Sylgard (Dow Corning), and heat-polished to a resistance of 8 to 10 mega ohms.

To record single-channel currents, an Axopatch 200B patch-clamp amplifier (Molecular Devices) was used with the gain set at 100 mV/pA and the internal Bessel filter at 10 kHz. After forming a giga-ohm seal, a continuous command voltage of +70 mV was applied to the interior of the patch pipette to establish a membrane potential of −70 mV. The resulting analog current recording was digitized at 20 μs intervals using a National Instruments model BNC-2090 A/D converter equipped with a PCI 6111e acquisition card and recorded to a PC hard disk using the program Acquire (Bruxton Corporation).

### Analysis of single-channel currents

Analysis of single-channel currents was conducted using TAC 4.2.0 (Bruxton Corporation). A digital Gaussian filter of 5 kHz within TAC was applied to the data. Single-channel events were detected using the half-amplitude threshold criterion as described (21). Dwell time histograms were plotted using a logarithmic *abscissa* and a square root ordinate (22) with an imposed dead time of 40 μs, and a minimum number of exponential components was fitted to the histograms by maximizing the likelihood using TACFit 4.2.0.

### Analysis of channel reopening

The probability of a channel that just closed will reopen was quantified by plotting the fraction of channel opening episodes with greater than N reopening events against the number of reopening events per episode. A channel opening episode, or burst, was defined as a series of one or more openings separated by closings briefer than a specified time τ_crit_ obtained from the point of intersection between the major long closed time component and that of the succeeding briefer component, as described (23); for a typical recording τ_crit_ ranged from 0.4 to 1 ms. A monoexponential decay function was fitted to the reopening plot yielding a decay constant in units of reopenings/per burst. An F-test was used to determine whether the fitted decay constant differed significantly between recordings of WT and mutant receptors, with significance defined as *p* < 0.05. Both the exponential fitting and the F-test were carried out using the Prism software package (GraphPad Prism 9.2.0; RRID:SCR_002798; https://www.graphpad.com).

### Burst duration

Bursts of channel openings were defined as a series of closely spaced openings flanked by closed intervals longer than the specified critical time τ_crit_ described above for analysis of channel reopening. The duration of a burst is thus the sum of the durations of the openings plus the intervening closings shorter than τ_crit_. The burst duration for WT or a given mutant receptor was the average of the mean burst durations from three patches obtained from a different cell and transfection under identical experimental conditions. Mean burst durations, with N = 3 for each mutant, are expressed as mean ± SD and analyzed by one-way ANOVA, followed by Dunnett’s multiple comparison post hoc test using GraphPad Prism 9.2.0.

### Single-channel current amplitude

For a given continuous sweep of single-channel data obtained at a membrane potential of −70 mV, a histogram of the digital points was generated, and two Gaussian functions were fitted to the data—one for baseline and the other for open channel current levels. The difference between the mean values of each Gaussian component was calculated and averaged for 45 to 60 sweeps to obtain the mean current amplitude for WT or a given mutant receptor.

### Radio-ligand binding

Cells in 10 cm tissue culture plates were maintained in Dulbecco's modified Eagle's medium (Gibco) with 10% fetal bovine serum, and when they reached 50% confluency, they were transfected with the desired cDNAs, as described for the single-channel recording experiments. To measure receptor expression, cells were harvested in PBS, centrifuged at 2500 rpm for 1 min, and resuspended in extracellular solution (mM): 140 KCl, 5.4 NaCl, 1.8 CaCl_2_, 1.7 MgCl_2,_ and 10 Hepes, with the pH adjusted to 7.4 with NaOH. Cell suspensions were divided into equal aliquots and incubated with 2 nM ^125^I-epibatidine (generously supplied by Dr Vanda Lennon, Neuroimmunology Laboratory, Mayo Clinic) for 30 min at 22 °C with or without preincubation with 10 mM ACh. Cell suspensions were filtered using a Brandel M-48T cell harvester (Gaithersburg, MD, USA) and radio-ligand bound to the cells was measured using a γ-counter. An identical protocol was applied to cells transfected with cDNA encoding the β2 subunit to measure nonspecific binding, which was subtracted from all measurements.

## Data availability

The data that support the findings of this study are available from the corresponding author upon reasonable request.

## Supporting information

This article contains Supporting information.

## Conflict of interest

The authors declare that they have no conflicts of interest with the contents of this article.
