# Supplementary material for: Pathogenic residue insertion in neuronal nicotinic receptor alters intra- and inter-subunit interactions that tune channel gating
Source: J Biol Chem. 2024 Apr 6;300(5):107266. doi: 10.1016/j.jbc.2024.107266 (PMC11067541; doi:10.1016/j.jbc.2024.107266)
Supplement: Supplementary Tables S1 and S2 [file mmc1.pdf]

Table S1: Single channel current amplitude for residue insertions and substitutions

Position of inserted residue in α4	Residue	Receptor-type	Current amplitude (pA) at -70 mV; Mean (95% CI); N=number of openings	Statistical difference between wild type and mutant	Fold change for mutant relative to wild type
	Wild type	$(\alpha 4)_2(\beta 2)_3$	2.40(2.37-2.44), N=53	-	-
259-260	Leu	$(\alpha 4^{259Leu260})_2(\beta 2)_3$	1.97(1.94-2.01), N=53	<0.0001	0.82
260-261	Leu	$(\alpha 4^{260Leu261})_2(\beta 2)_3$	1.84(1.79-1.82), N=54	<0.0001	0.77
	Ile	$(\alpha 4^{260Ile261})_2(\beta 2)_3$	1.81(1.79-1.83), N=63	<0.0001	0.75
	Ala	$(\alpha 4^{260Ala261})_2(\beta 2)_3$	2.11(2.06-2.16), N=54	<0.0001	0.88
261-262	Val	$(\alpha 4^{261Val262})_2(\beta 2)_3$	1.83(1.79-1.86), N=54	<0.0001	0.76
	Ala	$(\alpha 4^{261Ala262})_2(\beta 2)_3$	1.78(1.70-1.86), N=45	<0.0001	0.74
substituted residue in α4 261	Leu	$(\alpha 4^{Thr261Leu})_2(\beta 2)_3$	2.37(2.34-2.40), N=53	0.7993	0.99
	Ile	$(\alpha 4^{Thr261Ileu})_2(\beta 2)_3$	2.34(2.31-2.38), N=54	0.2978	0.98
	Val	$(\alpha 4^{Thr261Val})_2(\beta 2)_3$	2.40(2.36-2.44), N=54	>0.9999	1.00
	Ala	$(\alpha 4^{Thr261Ala})_2(\beta 2)_3$	2.45(2.41-2.50), N=53	0.5588	1.02

Table S2: Comparison of M2 side-chain orientation and functional consequences for GOF mutations

nAChR type	Original pathogenic mutation	M2 position	Side chain orientation	Prolonged open time	Spontaneous openings	Reference
Muscle	αV249F	7′	Intra-subunit	Yes	Yes	[1]
	βL262M	9′	Pore-facing	Yes	n.d.	[2]
	εT264P	12′	Inter-subunit (R)	Yes	Yes	[3]
	δS268F	12′	Inter-subunit (R)	Yes	n.d.	[4]
	αT254I	12′	Inter-subunit (R)	Yes	n.d.	[5]
	εV265A	13′	Pore-facing	Yes	n.d.	[6]
	βV266M	13′	Pore-facing	Yes	n.d.	[7]
	εL269F	17′	Inter-subunit (L)	Yes	n.d.	[7]
	δL273F	17′	Inter-subunit (L)	Yes	n.d.	[8]
Neuronal	α4S246F	6′	Inter-subunit (L)	n.d.	n.d.	[9]
	α4S250W	10′	Inter-subunit (L)	Yes	Yes	[10]
	**α4^{259Leu260}**	**18′**	**Intra-subunit**	**Yes**	**No**	**Present study**

Intra-subunit indicates the side chain is buried in the protein interior; Inter-subunit indicates the side chain projects against the neighboring subunit, R for right, L for left, when viewed from the extracellular side; Pore-facing indicates the side chain projects into the pore. Reference structure is the *Torpedo* nAChR with α-bungarotoxin bound (PDB: 6UWZ) [11]. M2 numbering begins on the intracellular side with the -1′ position corresponding to the Glu/Gln residue that forms the ion selectivity filter. Not determined, n.d.

1. Milone, M., et al., *Slow-channel myasthenic syndrome caused by enhanced activation, desensitization, and agonist binding affinity attributable to mutation in the M2 domain of the acetylcholine receptor alpha subunit.* J Neurosci, 1997. **17**(15): p. 5651-65.
2. Gomez, C.M., et al., *A beta-subunit mutation in the acetylcholine receptor channel gate causes severe slowchannel syndrome.* Ann Neurol, 1996. **39**(6): p. 712-23.

3. Ohno, K., et al., *Congenital myasthenic syndrome caused by prolonged acetylcholine receptor channel openings due to a mutation in the M2 domain of the epsilon subunit.* Proc Natl Acad Sci U S A, 1995. **92**(3): p. 758-62.
4. Gomez, C.M., et al., *Novel delta subunit mutation in slow-channel syndrome causes severe weakness by novel mechanisms.* Ann Neurol, 2002. **51**(1): p. 102-12.
5. Croxen, R., et al., *Mutations in different functional domains of the human muscle acetylcholine receptor alpha subunit in patients with the slow-channel congenital myasthenic syndrome.* Hum Mol Genet, 1997. **6**(5): p. 767-74.
6. Ohno, K., et al., *Slow-channel myasthenic syndrome caused by a novel mutation in the acetylcholine receptor ε-subunit*. Neurology, 1998. **50**: A432.
7. Engel, A.G., et al., *New mutations in acetylcholine receptor subunit genes reveal heterogeneity in the slow-channel congenital myasthenic syndrome.* Hum Mol Genet, 1996. **5**(9): p. 1217-27.
8. Shen, X.M., et al., *Slow-channel myasthenia due to novel mutation in M2 domain of AChR delta subunit.* Ann Clin Transl Neurol, 2019. **6**(10): p. 2066-2078.
9. Steinlein, O.K., et al., *A missense mutation in the neuronal nicotinic acetylcholine receptor alpha 4 subunit is associated with autosomal dominant nocturnal frontal lobe epilepsy.* Nat Genet, 1995. **11**(2): p. 201-3.
10. Mazzaferro, S., et al., *Genetic Variant in Nicotinic Receptor alpha4-Subunit Causes Sleep-Related Hyperkinetic Epilepsy via Increased Channel Opening.* Int J Mol Sci, 2022. **23**(20).
11. Rahman, et al., *Structure of the Native Muscle-type Nicotinic Receptor and Inhibition by Snake Venom Toxins.* Neuron, 2020. 1**06**(6): p. 952-962.
